# A highly sensitive and specific system for large-scale gene expression profiling

**DOI:** 10.1186/1471-2164-9-9

**Published:** 2008-01-10

**Authors:** Guohong Hu, Qifeng Yang, Xiangfeng Cui, Gang Yue, Marco A Azaro, Hui-Yun Wang, Honghua Li

**Affiliations:** 1 Department of Molecular Genetics, Microbiology and Immunology/The Cancer Institute of New Jersey, University of Medicine and Dentistry of New Jersey Robert Wood Johnson Medical School, Piscataway, New Jersey 08854, USA

## Abstract

**Background:**

Rapid progress in the field of gene expression-based molecular network integration has generated strong demand on enhancing the sensitivity and data accuracy of experimental systems. To meet the need, a high-throughput gene profiling system of high specificity and sensitivity has been developed.

**Results:**

By using specially designed primers, the new system amplifies sequences in neighboring exons separated by big introns so that mRNA sequences may be effectively discriminated from other highly related sequences including their genes, unprocessed transcripts, pseudogenes and pseudogene transcripts. Probes used for microarray detection consist of sequences in the two neighboring exons amplified by the primers. In conjunction with a newly developed high-throughput multiplex amplification system and highly simplified experimental procedures, the system can be used to analyze >1,000 mRNA species in a single assay. It may also be used for gene expression profiling of very few (*n *= 100) or single cells. Highly reproducible results were obtained from duplicate samples with the same number of cells, and from those with a small number (100) and a large number (10,000) of cells. The specificity of the system was demonstrated by comparing results from a breast cancer cell line, MCF-7, and an ovarian cancer cell line, NCI/ADR-RES, and by using genomic DNA as starting material.

**Conclusion:**

Our approach may greatly facilitate the analysis of combinatorial expression of known genes in many important applications, especially when the amount of RNA is limited.

## Background

Biological processes are underlain by interactions between various genes and their products through defined pathways in the molecular network, in which molecules cross communicate in hitherto unknown ways under both healthy and disease conditions. Learning gene expression patterns on a genomic scale would substantially help deconvolute these complex processes. Exhaustive identification of human genes during the Human Genome Project has made such studies possible. By global gene expression profiling in cells and tissues under either physiological or *in vitro *conditions, our understanding of the correlation between gene functions and their phenotypic effects could be significantly enhanced.

The advent of the microarray-based high-throughput RNA detection system [1,2] has made it possible to profile gene expression patterns for the entire transcriptome. However, to detect gene transcripts very specifically, one needs to discriminate them from closely related sequences including: (1) the corresponding gene sequences. Although contamination of gene sequences may not be a concern for applications using purified mRNA, gene sequences must be taken into consideration for applications directly using cell lysate without RNA extraction. This becomes especially important when the studied transcripts are present at low abundance; (2) pseudogenes and their possible transcripts. The number of pseudogenes in the human genome was estimated to be 20,000 to 33,000, which are widely expressed [3,4]. These sequences usually share a high degree of sequence identity with the closely related genes; (3) unprocessed RNA containing the same exons as those of the corresponding mRNA. So far, no system has addressed the above issue very effectively.

Among the microarray-based platforms, GeneChip is a commonly used system and has been improved significantly since it was invented, and has contributed to understanding the complex gene expression network in a great deal. However, since this technology is limited by its high degree of nonspecificity and insensitivity, its application has been limited in molecular network integration. Results from a recent analysis [5] indicated that on the Affymetrix GeneChip U95A/Av2 array, 20,696 (10.5%) probes were nonspecific, which could cross-hybridize to multiple genes, and 18,363 (9.3%) probes missed the target transcript sequences. The numbers of nonspecific and mis-targeted probes on the U133A array were comparable, which were 29,405 (12.1%) and 19,717 (8.0%), respectively [5]. These ~20% of problematic probes certainly and substantially compromise the data accuracy, decrease the value of microarray data, and are not acceptable for the studies of molecular network integration. It was also found that some probe sets representing the same genes on Affymetrix microarrays could show significant discrepancy because of the non-specific hybridization [6,7]

In most applications, gene expression profiling with microarrays including GeneChip requires amplification of sample RNA, regardless of how much material is available. Normally, 1 to 3 μg of RNA is required for each assay [8]. However, high-throughput gene expression profiling with superior sensitivity is becoming more and more demanded, and has its wide applications. For example, in breast cancer research, analysis of specimens from microdissection may provide important information about genes involved in different cancer development stages and for understanding the molecular mechanisms underlying cancer development [9]. Specimens from fine needle biopsy are also important in diagnostic procedures and in evaluating therapeutic effects. The ability to analyze a large number of genes in single cells may help understand the origin and clonality of cancer development and learn the molecular details involved in different stages of the cell cycle.

Current methodologies for gene expression profiling in small RNA samples, especially those from single cells, are very limited. Many of these protocols [2,10,11] require multiple enzymatic reactions that may seriously reduce the sensitivity and compromise the specificity. RNA preparation in most of applications also involves a number of steps, which is rather lengthy, tedious, and requires highly skilled personnel.

To solve the above problems, we have developed a highly specific and sensitive gene expression profiling system. With this system, primers are specially designed to amplify mRNA sequences very specifically. Probes used for microarray detection are designed only to hybridize to sequences amplified from mRNA. In conjunction with the high-throughput multiplex amplification protocol developed in our laboratory lately [12], a large number of mRNA species directly released from very few cells or even single cells can be amplified to a detectable amount without RNA isolation. Amplified products can then be detected by the single-base extension[13] assay on an oligonucleotide microarray [14].

## Results

### Experimental system used in the study

To establish a cancer gene expression array, a panel of cancer-related genes were selected based on their known functions and/or cancer-associated expression patterns from published literature [15-28]. All amplicon sequences were subjected to computational screening to ensure their uniqueness. Primers and probes were selected according to a series of criteria as specified in Materials and Methods. Most primer pairs amplify sequences in two neighboring exons separated by large introns. The intron lengths ranged from 79 bp to 90 kb with an average of 2.0 kb and 97% of the introns are longer than 200 bp. Initially 1,445 genes were used as the input for the primer and probe design program. Primers and probes were selected for 1,120 (77.5%) of these genes. The remaining 22.5% had either no introns or no suitable sequences for primers and/or probes. Fifteen of these remaining genes with important functions in cancer development were included in the panel. Primers and probes were designed based on the unique sequences in these genes, and were not required to have introns internally located within the amplified sequences. Therefore, a total of 1,135 genes were included in our multiplex assay. (Details about these genes, and their corresponding primers and probes used for the study are listed in Additional files 1 and 2.)

**Figure 1:**
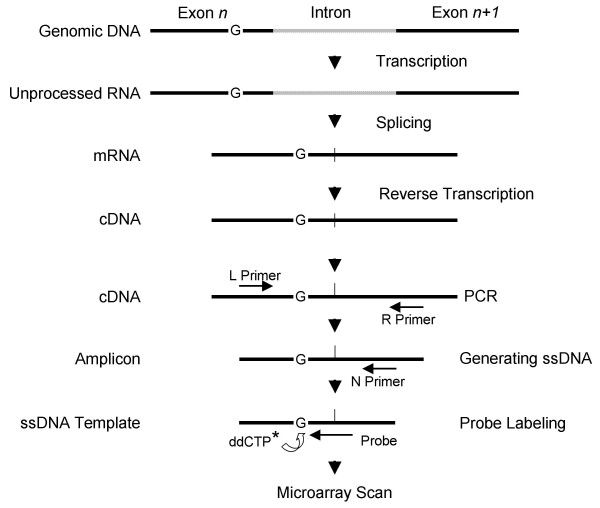
**Schematic illustration of the high-throughput gene expression profiling procedure**. Fluorescent labeling is indicated by an asterisk.

Microarray-based single-base extension (SBE) assay has been used to genotype single nucleotide polymorphisms (SNPs) [12,29,30] in our laboratory. In the present study, SBE was adapted for gene expression profiling. To simplify the analysis, all probes were designed to terminate immediately before a 'G' base in the templates. In this way, the probes were extended by a single base, dideoxynucleoside triphosphate (ddCTP) that was fluorescently labeled. By using one color, the bias associated with different dyes was also eliminated. The detection procedure is schematically illustrated in Fig. 1. Resulting data have been deposited to the NCBI's Gene Expression Omnibus (GEO) [31] and are accessible through GEO Series accession number GSE5920.

### Reproducibility of the high-throughput gene expression profiling system

**Figure 2:**
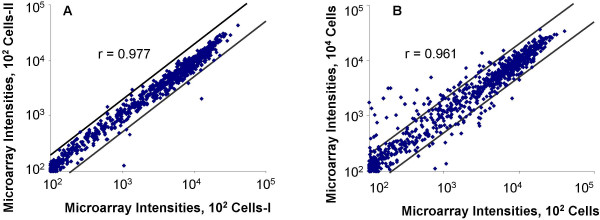
**Comparison of microarray results between duplicated samples**. **A**. A scatter plot of the microarray intensities of a sample with 100 NCI/ADR-RES cells were plotted against those of a duplicated 100-cell sample, and **B**, a scatter plot in **A **after replacing the data of the latter 100-cell sample with data of a 10,000-cell sample from the same cell line. Microarray intensities shown in the figure are the results after baseline subtraction. Both plots are on the log scale.

To test the reproducibility of our system, gene expression was profiled for three duplicated 100-cell samples from an ovarian cancer cell line, NCI/ADR-RES [32] and two 100-cell samples from a breast cancer cell line, MCF-7. Resulting microarray data are supplied in Additional file 3. Table 1 summarizes the numbers of gene transcripts detected from different samples. As shown, 660 (58.2%), 663 (58.4%), and 662 (58.3%) gene transcripts were detected from the three 100-cell samples of NCI/ADR-RES, respectively. Of these transcripts, 650 (>98%) were detected from all three duplicates. Signal intensities for the 1,135 genes were strongly correlated between the duplicates (Pearson's r = 0.977, 0.974, and 0.949, respectively). Fig. 2A shows a scatter plot of two duplicates. Of the 650 transcripts detected in all three NCI/ADR-RES 100-cell samples, only 6 (0.9%), 17 (2.6%), and 1 (0.2%) transcripts had their signal intensities differing by >2 fold between each two of these three duplicates. Twenty-six transcripts were detected from only one or two of the three samples. The signal intensities for these transcripts were low. Only one transcript in one sample had its signal intensity >1,000, indicating that the inconsistence among the duplicates was due to low signals of these transcripts.

**Table 1:** Detection of the 1,135 gene transcripts in various samples

**Cell Line**	ADR*							**MCF-7**	
		
	**Single-Cell**			**100-Cell**			**10,000-Cell**	**100-Cell**	
				
**Sample**	**I**	**II**	**III**	**I**	**II**	**III**		**I**	**II**
				
**Detectable**	590	576	614	660	663	662	655	615	614
				
**Undetectable**	545	559	521	475	472	473	480	520	521
				
**Detectable in all**	504			650				597	
				
**Detectable in some**	182			26				35	
				
**Undetectable in all**	449			459				503	
				
**Detectable in all**				630					
			
**Detectable in some**				63					
			
**Undetectable in all**				442					
			
**Detectable in all**					531					
		
**Detectable in some**				212					
		
**Undetectable in all**				392					
		
All ADR*, not MCF-7				75					
		
Both MCF-7, not ADR*				43					
		
**Detectable in all**	463								
		
**Detectable in some**	315								
		
**Undetectable in all**	357								
		
**Detectable in all non-single, not in single**	61								
		
**Detectable in all single, not in non-single**	27								

*ADR: NCI/ADR-RES

For the two 100-cell samples from MCF-7, 615 (54.2%) and 614 (54.1%) gene transcripts were detected, respectively, with 597 (>97%) detected in both. Of these 597 transcripts, 562 (94.1%) had signal intensities differing less than two fold. Similar to the situation with NCI/ADR-RES samples, all 34 transcripts that were detected in only one sample but not the other had low signal intensities with only nine genes whose signal intensities were >1,000 in one of the two samples.

Because samples prepared from a large number of cells are usually associated with high reliability, we further compared the microarray results of the NCI/ADR-RES 100-cell samples with those from a 10,000-cell sample of the same cell line. Resulting data also show a high degree of correlation (r = 0.961, Figure 2B). As shown in Table 1, 630 (96.7%) of the 650 gene transcripts detected from all the 100-cell samples were also detected from the 10,000-cell sample. Sixty-three gene transcripts were detected in at least one of the three 100-cell samples but not in the 10,000-cell sample, or *vice versa*. Of these 63 gene transcripts, 61 had signal intensities below 1,000 in all the three 100-cells. However, the change from 100 to 10,000 cells did enhance the detection of 21 gene transcripts whose signal intensities were >2 fold greater in the 10,000-cell sample than those in the 100-cell samples. Among these 21 transcripts, six had signal intensities in the 10,000-cell sample more than 15 fold greater than the average intensities of the corresponding genes in the three 100-cell samples, indicating that using 10,000 cells may have significantly increased the copy numbers of these transcripts or changed their absence status to presence. These data indicate that our system not only can produce very reliable results even with as few as 100 cells but also is very sensitive to the copy number change for the low-copy-number gene transcripts.

### Sensitivity of the high-throughput gene expression profiling system

To further test the sensitivity of our high-throughput gene expression profiling system, single NCI/ADR-RES cell samples were prepared and used for multiplex gene expression assay of the 1,135 mRNA species. Microarray results from three of these samples are listed in Additional file 3. The numbers of gene transcripts detected from the three single-cell samples were 590, 576, and 614, respectively. Of these transcripts, 503 were detected from all single cells. Of the 503, 463 (92.0%) were also detected from all non-single-cell (100-cell and 10,000-cell) samples, indicating a prevalent expression of these genes in most, if not all, cells at relatively high levels.

On the other hand, the detection range of gene transcripts from the three single-cell samples was wider compared to the non-single-cell samples. As shown in Table 1, 449 transcripts were undetectable in all three single cell samples, a number which is not greater than that (459) for the three 100-cell samples and is comparable to that (442) for all non-single-cell samples. The number of undetectable gene transcripts from all single and non-single cell samples is 357. This number means that from single cells, we not only detected a comparable number of genes, but also detected a new set of 449-357 = 92 genes that could not be detected with non-single-cell samples of the same cell line.

The robustness of gene expression profiling with single-cell samples was also demonstrated by the signal intensities. As described above, most transcripts that were detected from some but not all non-single-cell samples had low signal intensities and very few were >1,000. The scenario with single cells is very different. Of the 503 gene transcripts detected from all single cells, 40 were detected in one to three non-single-cell samples but not all four. All 40 but one have signal intensity >1,000 in at least one of the three single-cell samples. Of the 183 transcripts that were only detected from one or two single-cell samples, 108 (59.0%) had signal intensity >1,000. The strong and robust signal intensities detected from single-cell samples indicate that our system is very sensitive.

Unlike the gene transcripts detected from all non-single-cell samples which account for more than 95% of gene transcripts detected from each of these samples, the 503 gene transcripts detected from single cells only account for 85. 3%, 87.3% and 81.9% of the transcripts detected from individual single-cell samples, respectively. Pairwise comparison of the results from the single-cell samples yielded correlation coefficients of 0.780, 0.700, and 0.711, respectively, compared with = 0.949 for the non-single-cell samples. From all single and non-single-cell samples, 778 gene transcripts were detected, of which 315 (40.5%) were detected from some but not all samples. This is in contrast with the scenario of non-single-cell samples from which gene transcripts that were only detected from some but not all samples were a very small portion (Table 1). Furthermore, of these 315 transcripts, 177 (56.2%) were either detected from only single cells or from non-single cell samples.

The high degree of concordance among the results from the non-single-cell samples, and the significant differences among those from single cells, and between single cells and non-single-cell samples indicate that most, if not all, of these differences are real. As mentioned above, this is further supported by the robustness of the signal intensities detected from single-cell samples for the gene transcripts that were detected differently between the single cells and non-single-cell samples. It is conceivable that heterogeneity in clonality and/or genetic alterations in the cells of a cell line could be major factors contributing to the differences. In addition, a considerable portion of the cells may be at different cycle stages during which groups of genes are expressed differently. Therefore, while gene expression in single cells could differ in various aspects, 100 cells may well represent the entire cell population because, after all, the cell line cells are from the same tissue and the same donor. Therefore, genes that are detectable in a cell population may not be expressed or expressed at very low levels in certain single cells. Conversely, genes that are detectable in particular single cell samples may not be expressed or expressed at very low levels in the majority of the cell population.

### Differential gene expression in the two cell lines, NCI/ADR-RES and MCF-7

When the gene expression profiles of NCI/ADR-RES were compared with those of MCF-7, a considerable number of genes were shown to be expressed differentially in these two cell lines. Of the 1,135 gene products, 531 (46.8%) were detected from samples of both cell lines (not including single cell samples). Seventy-five gene transcripts were detected in all NCI/ADR-RES non-single-cell samples, but not in the MCF-7 samples, and 43 were detected in the opposite way.

**Figure 3:**
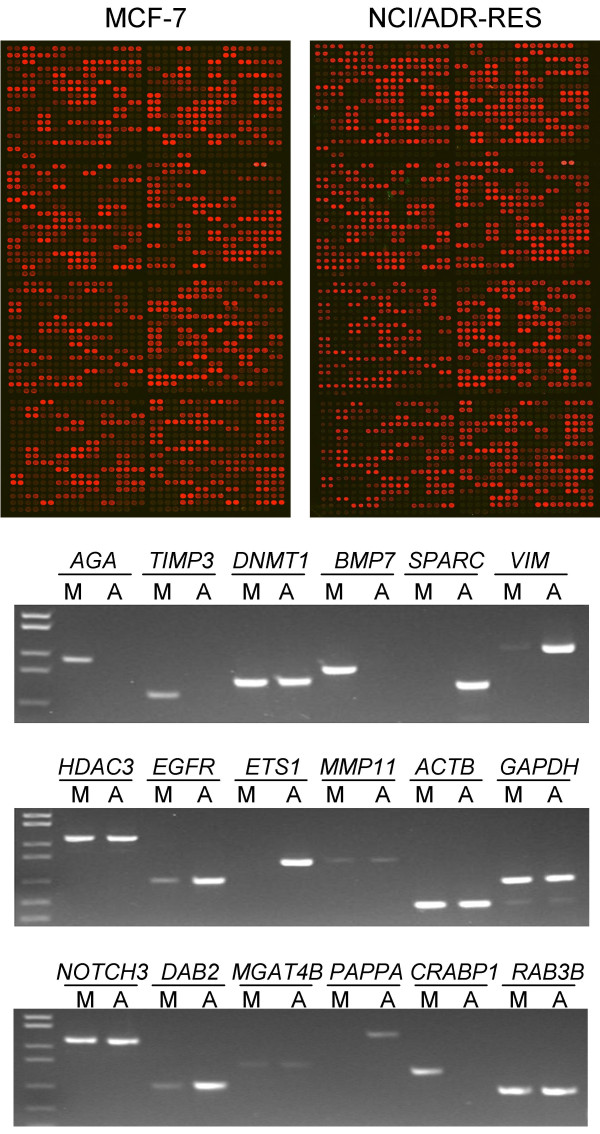
**Differential gene expression in the two cell lines, MCF-7 and NCI/ADR-RES**. **Upper**, Microarray images from the two cell lines. **Lower**, confirmation of the gene expression profiling results by gel assay. Quantitative results are given in Table 3. M, MCF-7, and A, NCI/ADR-RES.

Of the 118 differentially expressed genes, 69 were shown to be expressed with more than 10-fold difference (Table 2). Of the 69 genes, 37 (53.6%) were detected as strongly or relatively strongly expressed in MCF-7, but weakly or not expressed in NCI/ADR-RES, and 32 were detected in the opposite way. To validate the gene expression data, 22 of these 69 genes, and another 46 gene transcripts detected with various microarray signal intensities different between the samples of the two cell lines were randomly selected (Table 3) and subjected to RT-PCR amplification individually. The amplified products were resolved by gel electrophoresis. The signal intensities of the respective bands were quantified with a gel documentation system. Part of results from microarrays and gel assays are shown in Fig. 3.

**Table 2:** Differentially expressed genes in the two cell lines, MCF-7 and NCI/ADR-RES

**Category**	**Gene Ref#**	**Probe ID**	**Gene Symbol**	**Log_2 _Ratio (NCI/ADR-RES:MCF-7)**
Overexpressed genes in NCI/ADR-RES	NM_004126	BYH0575	GNG11	6.69
	NM_004355	BYH0596	CD74	6.38
	NM_005822	BYH0767	DSCR1L1	6.20
	NM_005238	BYH0702	ETS1	6.06
	NM_002019	BYH0339	FLT1	5.99
	NM_000110	BYH0058	DPYD	5.81
	NM_001792	BYH0302	CDH2	5.65
	NM_001336	BYH0239	CTSZ	5.63
	NM_024423	BYH1071	DSC3	5.60
	NM_001250	BYH0223	CD40	5.51
	NM_001511	BYH0257	CXCL1	5.36
	NM_000873	BYH0185	ICAM2	5.32
	NM_032727	BYH1080	INA	5.09
	NM_005465	BYH0738	AKT3	4.98
	NM_003118	BYH0466	SPARC	4.94
	NM_001561	BYH0267	TNFRSF9	4.91
	NM_000576	BYH0143	IL1B	4.62
	NM_003644	BYH0527	GAS7	4.62
	NM_001953	BYH0328	ECGF1	4.62
	NM_001839	BYH0312	CNN3	4.57
	NM_033293	BYH1093	CASP1	4.47
	NM_015873	BYH0979	VILL	4.47
	NM_057162	BYH1100	KLHL4	4.39
	NM_021913	BYH1052	AXL	4.33
	NM_000700	BYH0168	ANXA1	4.23
	NM_000584	BYH0145	IL8	4.18
	NM_000088	BYH0052	COL1A1	4.10
	U28727	BYH1107	PAPPA	4.00
	NM_001150	BYH0210	ANPEP	3.62
	NM_003659	BYH0530	AGPS	3.61
	NM_006097	BYH0797	MYL9	3.43
	NM_012449	BYH0928	STEAP1	3.35

Underexpressed Genes in NCI/ADR-RES	NM_001789	BYH0300	CDC25A	-3.34
	NM_014427	BYH0960	CPNE7	-3.38
	NM_004708	BYH0646	PDCD5	-3.53
	NM_005235	BYH0700	ERBB4	-3.63
	NM_001702	BYH0281	BAI1	-3.68
	NM_005568	BYH0743	LHX1	-3.70
	NM_005994	BYH0787	TBX2	-3.78
	NM_033016	BYH1086	PDGFB	-3.83
	NM_001759	BYH0291	CCND2	-3.89
	NM_006180	BYH0803	NTRK2	-3.90
	NM_000090	BYH0054	COL3A1	-4.00
	NM_002051	BYH0343	GATA3	-4.18
	NM_001422	BYH0247	ELF5	-4.24
	NM_000429	BYH0117	MAT1A	-4.32
	NM_000633	BYH0158	BCL2	-4.57
	NM_000949	BYH0193	PRLR	-4.59
	NM_014333	BYH0955	CADM1	-4.79
	NM_002555	BYH0402	SLC22A18	-5.00
	NM_003722	BYH0533	TP73L	-5.01
	NM_021111	BYH1039	RB1	-5.09
	NM_012116	BYH0906	CBLC	-5.15
	NM_000550	BYH0139	TYRP1	-5.17
	AF055033	BYH0006	IGFBP5	-5.52
	X52599	BYH1117	NGFB	-5.53
	NM_000027	BYH0029	AGA	-5.54
	M35410	BYH0025	IGFBP2	-5.59
	NM_003486	BYH0521	SLC7A5	-5.75
	NM_018684	BYH1015	KIAA1166	-6.08
	NM_000362	BYH0102	TIMP3	-6.14
	NM_004378	BYH0600	CRABP1	-6.79
	NM_005410	BYH0726	SEPP1	-6.79
	NM_001719	BYH0284	BMP7	-6.83
	NM_003282	BYH0499	TNNI2	-7.06
	NM_004048	BYH0570	B2M	-7.07
	NM_003177	BYH0473	SYK	-7.18
	NM_004561	BYH0622	OVOL1	-7.20
	NM_000609	BYH0154	CXCL12	-8.3

**Table 3:** Comparison between the results from microarray and gel assay*

		**Microarray Intensity**		**Gel Assay Intensity**		**Ratio ADR/MCF-7**	
		
**Gene Group**	**Gene Symbol**	**ADR**	**MCF-7**	**ADR**	**MCF-7**	**Micro-array**	**Gel Assay**
**I**	BAI1	1,192	12,372	4,578	1,320	0.10	3.47
	SSTR2	9,206	19,108	2,887	7,684	0.48	0.38
	MCL1	17,027	31,012	7,481	8,901	0.55	0.84
	TPM2	8,582	14,489	34,703	13,186	0.59	2.63
	ACOX1	8,125	13,350	26,206	19,508	0.61	1.34
	MGAT4B	2,274	3,219	1,053	922	0.71	1.14
	UBQLN1	27,113	38,083	24,037	20,089	0.71	1.20
	RAB3B	28,188	31,908	33,571	31,044	0.88	1.08
	DNMT1	25,315	27,675	41,164	43,718	0.91	0.94
	BUB1B	13,233	14,452	21,197	14,321	0.92	1.48
	PRDM10	13,233	14,452	9,419	6,641	0.92	1.42
	NOTCH3	7,187	7,618	26,090	24,176	0.94	1.08
	YY1	27,020	27,930	40,692	33,876	0.97	1.20
	TPR	13,081	13,176	32,959	26,423	0.99	1.25
	PTTG1IP	12,506	12,298	15,621	12,424	1.02	1.26
	PPP2R4	16,105	15,740	15,593	19,497	1.02	0.80
	YES1	21,006	20,434	18,228	14,830	1.03	1.23
	ZNF670	23,207	22,487	6,581	6,904	1.03	0.95
	RAP1GDS1	16,806	15,385	32,743	22,443	1.09	1.46
	CKS2	21,507	19,672	10,614	9,289	1.09	1.14
	HDAC3	26,309	22,572	28,826	29,169	1.17	0.99
	GAPDH	9,369	7,916	34,584	33,939	1.18	1.02
	PTTG1	12,802	10,790	18,680	16,447	1.19	1.14
	HSPA5	19,233	16,146	17,077	15,423	1.19	1.11
	ACTB	6,849	4,400	35,335	35,859	1.56	0.99
	ASPH	10,942	4,035	20,194	3,502	2.71	5.77
	S100A2	3,498	1,057	13,688	9,559	3.31	1.43
	TFG	13,571	3,915	14,015	15,954	3.47	0.88
	EGFR	6,841	1,676	34,449	7,738	4.08	4.45
	RELA	8,209	1,970	20,099	12,783	4.17	1.57
	VIM	12,172	2,192	48,836	2,518	5.55	19.39
	RTN1	8,770	1,133	17,159	2,008	7.74	8.55

**II**	CRABP1	94	8,594	0	21,529	0.01	0.00
	BMP7	92	8,825	0	43,366	0.01	0.00
	TIMP3	92	5,428	0	17,135	0.02	0.00
	AGA	90	3,457	0	20,178	0.03	0.00
	CXCL12	97	24,456	28	16,471	0.00	0.00
	B2M	98	10,621	990	13,599	0.01	0.07
	TYRP1	181	5,380	2,125	7,089	0.03	0.30
	PDGFB	352	4,080	2,460	6,627	0.09	0.37
	GATA3	176	2,618	2,780	4,343	0.07	0.64
	SEPP1	84	7,626	3,056	4,432	0.01	0.69
	TBX2	202	2,255	4,808	9,482	0.09	0.51
	HDAC5	243	1,266	16,605	7,834	0.19	2.12

**III**	ETS1	22,413	280	40,334	0.00	79.97	-
	SPARC	9,959	266	44,142	0.00	37.45	-
	DSC3	18,136	304	8,076	0.00	59.62	-
	CD40	17,887	319	15,499	0.00	56.04	-
	FLT1	16,379	209	4,032	371	78.23	10.87
	INA	18,499	438	7,908	411	42.27	19.23
	CASP1	8,146	298	17,307	447	27.34	38.72
	CDH2	12,318	200	10,259	477	61.58	21.53
	GNG11	26,835	213	21,000	576	126.28	36.49
	MYL9	4,007	302	27,023	1,017	13.26	26.57
	CITED1	996	253	9,542	2,606	3.93	3.66
	DAB2	1,568	297	32,458	5,136	5.28	6.32
	CTSZ	12,657	211	22,322	17,993	59.87	1.24

**IV**	GNRHR	92	214	27	0.00	0.43	-
	STK32B	82	208	0.00	241	0.39	0.00
	IL13	82	191	0.00	464	0.43	0.00
	CDH5	88	213	0.00	519	0.41	0.00
	IL3	95	203	86	162	0.47	0.53
	LYL1	85	218	128	64	0.39	2.01
	CA1	87	192	365	95	0.45	3.83
	TYROBP	91	199	487	169	0.46	2.89
	IL13RA2	87	187	1,771	433	0.47	4.09
	IRF8	86	212	3,695	571	0.41	6.47
	MMP11	145	234	3,060	2,308	0.62	1.33

Note: 1. Signal intensities of microarray are averages of those respective non-single cell samples. 2. ADR: NCI/ADR-RES.

Table 3 summarizes the results from both microarray and gel assays. Based on the results from microarray, genes in Table 3 are subdivided into four groups. Transcripts of Group I genes were detected from all samples, while no transcripts were detectable from all samples for Group IV. Transcripts of Group III genes were detected only from the NCI/ADR-RES samples but not from the MCF-7 samples, and those of Group II genes were detected in an opposite way. As shown, the signal ratios between NCI/ADR-RES and MCF-7 from microarray for the Group I genes are well in concordance with the ratios from gel assay, with a correlation coefficient of 0.679, indicating results from microarray and gel assays match very well. Because signals of genes in Groups II to IV were below the background for at least one of the two cell lines, ratio comparison may not be meaningful. However, it is clear that the microarray signals detected from MCF-7 are all greater than those from NCI/ADR-RES for the Group II genes, and *vice versa *for the Group III genes. Results from the gel assay are very well in concordance with this correlation. The only exception is HDAC5 whose microarray signal from MCF-7 is approximately 5 times that from NCI/ADR-RES, while its gel signal is more than 2 times that of the latter. Since the microarray signal intensity from MCF-7 for this gene is the lowest among the Group II genes, this discrepancy could be caused by wider variation of the low signal intensity. For all genes in Groups II to IV, if the microarray signals are lower than background, the corresponding gel signals are also low (<5,000) except for three genes, HDAC5 in Group II, and DAB2 and CTSZ in Group III. The fact that low or relatively low signals were detected by the gel assay for the genes whose array signals were weaker than background may be a reflection of the difference between the two assays. For microarray, all 1,135 transcripts were amplified in the same tube, while all transcripts analyzed by gel assay were amplified individually. It is known that during PCR, after the reaction reaches a saturation point, very little additional products may be generated. When the gene transcripts are amplified in a multiplex way, certain low-copy-number sequences may not be amplified to the detectable amounts when the reaction reaches a saturation point.

### Specificity of the high-throughput gene expression system

The specificity of our high-throughput gene expression system was demonstrated by the results from different cell line samples and by those from different single cells as described above. To further demonstrate the specificity of our system, human genomic DNA samples were amplified with the same multiplex RT-PCR procedure and analyzed by microarray. Very few probes (<0.2%) were shown to have signals above background (data not shown), indicating that our system is very specific and can discriminate between the target mRNA sequences from their genomic counterparts, and therefore, the unprocessed transcripts. Our previous experience also showed that in the absence of specific templates, a few primer sets may amplify non-specific sequences. However, such non-specific amplification may become undetectable in the presence of specific templates because the specific sequences are much stronger in competition. In addition, using specially designed probes also enhanced the specificity.

## Discussion

Compared with other existing gene expression profiling methods, our approach has the following advantages:

### (1) Highly specific

To date, no other high-throughput system has been reported to be highly discriminative of mRNA from other related DNA and RNA sequences. Using primers amplifying sequences across intron(s) and probes consisting of sequences in adjacent exons is a critical enhancement to achieve such high specificity. Furthermore, all primer, probe and amplicon sequences were subjected to exhaustive searches against the databases of the entire human genome and transcriptome to ensure these sequences are unique. Such a step was proven very effective for enhancing the specificity [12]. Experimentally, when genomic DNA was used as samples, signals were only detected for only 2 or 3 genes (0.2%) out of the 1,135 genes. Based on our previous studies, these signals may become undetectable in the presence of specific sequences which may compete out the nonspecific amplification.

### (2) Highly sensitive

We showed previously that our multiplex amplification system could detect >1,000 single-copy sequences simultaneously from single haploid sperm cells [12]. The fact that >90% of these sequences are detectable indicates that with our specially designed primers, most, if not all, sequences may be well amplified parallelly with very limited, if any, interaction among the primers. Since the primers used for gene profiling are designed in the same way, it is reasonable to believe that most gene transcripts are also amplified parallelly. However, since the copy number of different gene transcripts in the cells varies in a wide range, the outcome of amplification would be different from that using single-copy sequences. When only single-copy sequences are used in multiplex amplification, most, if not all, sequences may reach the detectable amount before the system is saturated. However, when gene transcripts are amplified, whether a transcript reaches a detectable amount before the system is saturated depends on its copy number in the sample, and not all sequences may reach a detectable amount at the end of amplification. This is probably why some sequences were undetectable by microarray but detectable by gel assay.

With our system, a total of 686 gene transcripts were detected from three single cells, which is comparable to 676 for the three 100-cell samples and 693 for all non-single-cell samples from the same cell line. The sensitivity of our system is further proved by the facts that results from 100-cell samples are very similar to each other and to those from 10,000 cells, and that specific gene expression profiles were obtained from different cell lines using as few as 100 cells.

The sensitivity of our system is further illustrated by the results that a significant portion of transcripts that could not be detected from the NCI/ADR-RES samples but were detected from the MCF-7 samples or single cell samples, and *vice versa*. This also indicates that low microarray intensities for these transcripts were not false negatives, and they were either not present or present in very low abundance in the respective samples.

### (3) Very simple

Unlike other methods that involve multiple steps and use multiple enzymes, our method allows a large number of gene products amplified by a single RT-PCR step directly from cell lysates without RNA extraction. In this way, a large number of samples may be analyzed easily and cost-effectively. Our simple experimental procedure is also the basis of the high degree of sensitivity since it avoids complicated mRNA extraction and processing procedures before and during amplification, which may cause mRNA degradation or loss.

### (4) Very safe for RNA samples

When working with RNA, one has to take extra precaution to prevent mRNA from degradation. Our method does not need RNA extraction. Once cells are lysed, RNA is directly released to the RT-PCR buffer and used as template immediately. There is almost no chance for RNase to degrade the mRNA templates.

### (5) Highly flexible

Many studies may not need to analyze all genes in the human genome and may often need to focus on different gene groups. Therefore, flexibility of the experimental system would be highly desirable. With our computer program, a large number of gene products can be designed into a single multiplex group. Genes can be easily organized into different subgroups upon need, and can also be re-grouped at any time without altering the reaction conditions. New gene products can be added to an existing set easily.

The capacity of multiplex RT-PCR is another concern for high-throughput gene expression profiling because it not only makes the amplification of a large number of gene products affordable and cost-effective, but also eliminates challenges involved in quality control of RT-PCR for a large number of genes individually [33,34]. However, the capacity of multiplex amplification was limited by interaction between primers. A previous study reported a screening of 29 expressed genes using multiplex RT-PCR, but was unable to reduce the number of the reaction tubes less than eight [35]. Other studies multiplexed up to nine genes with nonspecific RT primers [36,37]. Studies using multiple sets of gene-specific primers in single reactions were also reported, but none of these generated enough products for the analysis of all expressed genes in the samples [34,38]. In the present study, we report our success with multiplex RT-PCR for 1,135 mRNA species. Such a success was based on a combination of several technological developments, including computerized primer design with predicted minimal interaction, a narrow primer T_m _range, small amplicon sizes, and optimization of amplification conditions based on our previous experience [12,29,30]. With our current protocol, it is possible to include two thousand or more gene transcripts in a single multiplex amplification group, and to analyze all human gene transcripts using several multiplexing amplification groups. After pooling amplified products from the multiplexing groups, all genes may be analyzed with a single microarray. With our system, large-scale gene expression profiling becomes highly affordable and cost-effective. If the primers and probes used in the high-throughput analysis are made accessible to the research community through a distribution system, large- and genome-scale gene expression profiling may be even more affordable and cost-effective.

A major limitation of our system is the requirement of presence of large introns in genes under study. When the introns are small, discrimination between mRNA and closely related DNA and RNA sequences is still possible by using probes consisting of sequences in the neighboring exons. For genes with no introns, primers and probes can be designed only to discriminate mRNA sequences from related pseudogenes and their transcripts but not the corresponding gene sequences. In this case, discrimination between mRNAs and their gene sequences is only possible when the mRNAs are present abundantly.

An extreme and possible application of our highly sensitive gene expression profiling system is the analysis of disseminated tumor cells in cancer research. Analysis of individual cells is necessary for understanding the early dissemination of tumor cells. Disseminated tumor cells remain in the patient bodies even after complete resection of the primary tumor, and can be obtained by bone marrow aspirates [39]. With our highly sensitive system, genetic signature in these cells may be detected. The resulting information may provide molecular basis for new therapeutic targets. For example, *ERBB2 *expression has been found to be a therapeutic target for metastatic breast carcinoma [40]. Identification of mRNA like that of *ERBB2 *in micrometastatic cells may help develop effective therapeutical approaches to preventing further development of these cells into incurable metastasis. Using mRNA from a small number of microdissected frozen tissue sections without RNA isolation has been demonstrated with a small number of genes [41]. Our system should be capable of using both microdissected and biopsy specimens for gene expression analysis on a much larger scale.

High-throughput gene expression profiling with single cells is also interesting for most laboratories studying molecular neurophysiology, but has been hampered by the capacity of multiplex PCR. Our approach can be used to examine the expression of many genes within individual neurons or other cells. The gene expression profiles can also be correlated to the phenotypes of these cells such as morphological, electrophysiological and pharmacological features to understand the underlying molecular mechanisms.

## Conclusion

This report describes a high-throughput gene expression profiling technology, which is simple, highly reproducible, specific and sensitive, and may greatly facilitate gene expression profiling of a small number of or even single cells. It may also be applicable to many applications where the amount of material is limited, and to diagnostic assays that identify the onset of cancer and monitor its progression, remediation or response to treatments.

Data discussed in this publication have been deposited in the NCBI's Gene Expression Omnibus [31] and are accessible through GEO Series accession number GSE5920.

## Methods

### Cell lines and single cell preparation

Human breast cancer cell line MCF-7 and ovarian cancer cell line NCI/ADR-RES were kindly provided by Drs. Jinming Yang, Hao Wu and William Hait [42]. The cell lines were maintained in RPMI 1640 medium containing 10% fetal bovine serum, 100 units/ml penicillin, and 100 μg/ml streptomycin at 37°C in a humidified atmosphere containing 5% CO_2_. After counting with a hemacytometer, cells were suspended in PBS (phosphate buffer solution) to 1000 cells/μl or other desirable densities. Two μl was dispensed into an Eppendorf tube containing cell lysis buffer (1.5 μl RNase inhibitor, 4 μl of 5× QIAGEN OneStep RT-PCR buffer, 12.5 μl H_2_O). Single cells were prepared from a diluted cell suspension of 2 cells/μl in 1 × PBS. About 0.5 μl of the suspension was pipetted onto a small piece of glass coverslip, and was checked under a microscope. If the droplet contained only one cell, the piece of the coverslip was then transferred into an Eppendorf tube containing the cell lysis buffer. The tube was immediately frozen in an ethanol/dry ice bath and stored at -80°C until use.

### Selection of genes for mRNA profiling

Genes used in the present study were selected based on previous publications [15-28], and are those involved in fundamental cell functions such as cell cycle, apoptosis, cell matrix, DNA repair, DNA replication, somatic recombination, RNA transcription and regulation, and protein translation and regulation. The borders between exons and introns for the selected genes were determined by aligning of the mRNA to genomic sequences using the BLAT program [43] maintained by the University of California, Santa Cruz.

### Primer and probe design

A computer program was written for primer and probe selection. Each pair of PCR primers was designed to amplify sequences in two adjacent exons flanking a large intron and to ensure specific amplification of the desirable mRNA sequences rather than the respective gene or unprocessed RNA sequences. To enhance the amplification specificity, the program always searches for candidate amplicon sequences separated by large introns in each gene. The melting temperatures (T_m_'s) for all selected primers ranged from 50.1°C to 61.6°C, and the GC-contents ranged from 32% to 70%. The lengths of the amplicons ranged from 72 to 150 bases.

Each oligonucleotide probe for microarray analysis was designed to consist of sequences of two adjacent exons to specifically interrogate the cDNA from corresponding mRNA sequence, but not the corresponding gene sequences or cDNA from unprocessed RNA. To facilitate microarray analysis, the 3'-ends of all probes terminated before a "G" base in the template sequence so that they can be labeled with the same fluorescent color by incorporating fluorescently labeled Cy5-ddCTP. The lengths of the probes ranged from 22 to 31 bases, and the GC-content of the probes ranged from 30% to 70% with their T_m_'s from 54.4°C to 65.2°C.

The BLAST executable program and sequence databases were downloaded from NCBI website [44] and installed to a local server. All the primers were subjected to BLAST search both in the human genome and the transcriptome databases to avoid amplification of nonspecific genomic or RNA sequences including pseudogenes and their RNA products. In addition, all primers and probes were subjected to interaction analysis with a computer program developed for designing high-throughput multiplex nucleotide acid detection [12]. Probes complementary to intron regions of some genes were also designed as negative controls. All amplicon sequences were subjected to BLAST search to ensure their uniqueness. Details about the primer and probe design for the high-throughput multiplex nucleic acid detection may be found in our previous publication [12].

### Gene-specific reverse transcription and multiplex RT-PCR

Cells in the lysis buffer described above were lysed with three repeating cycles of alternating one-min incubations from the ethanol/dry ice mix to a 37°C water bath before RT-PCR. One-step RT-PCR was carried out in a 50-μl reaction containing primers (20 nM each) for all the 1,135 mRNA species, 2.5 mM MgCl_2_, the four dNTPs (400 μM each), and 2.0 μl QIAGEN OneStep RT-PCR Enzyme Mix without degenerated primers. The samples were first incubated at 50°C for 40 min for cDNA synthesis, and then were heated to 95°C for 15 min to inactivate the reverse transcriptase and activate the *Taq *DNA polymerase followed by 45 PCR cycles. Each PCR cycle consisted of 40 sec at 94°C for denaturation, and 1 min at 55°C and 5 min of ramping from 55°C to 70°C for annealing and extension. A final extension step was carried out at 72°C for 3 min at the end of the PCR. All PCRs were performed with the PTC100 Programmable Thermal Controllers (MJ Research). Single-stranded DNA (ssDNA) was generated by using the same conditions in multiplex PCR except for the templates that were 10 μl of the multiplex RT-PCR product. Only one primer for each sequence was used, and 40 thermal cycles were carried out.

### RT-PCR with individual gene transcripts

RT-PCRs with individual gene transcripts were performed for a group of genes with different amounts of signal intensities detected from the two cell lines, NCI/ADR-RES and MCF-7. For each gene, an aliquot (equivalent of 100 cells) from the same cell lysate used for multiplex gene expression profiling was used. Conditions for one-step RT-PCR were similar to those for multiplex one-step RT-PCR. mRNAs transcribed from β-actin and α-tubulin genes served as internal controls. The PCR products were assayed by gel electrophoresis. Gels were imaged using an Image Station (Model 440, Kodak, New Haven, CT, USA). Gel band intensities were digitized with the software, Kodak 1D 3.5.

#### Microarray design, hybridization, and probe labeling by single-base extension assay

Oligonucleotide probes were printed onto glass slides in duplicate with a spot diameter of 160 μm and a center-to-center distance of 250 μm by using the OmniGrid Accent Microarrayer (Gene Machines, CA). One hundred fourteen spots with only microarray printing buffer without probes were used as negative controls and were distributed spatially evenly across each array. Microarray analysis was performed according to a four-step procedure established in our laboratory [12]. Briefly, (1) preparation of microarray slides: Pre-cleaned Gold Seal Micro slides (Becton Dickinson) with no scratch were chosen, and were soaked in 30% bleach with shaking for 1–2 hrs followed by rinsing five times with deionized H_2_O and three times with MilliQ H_2_O. Slides were then sonicated in 15% Fisher brand Versa-Clean Liquid Concentrate with heat on for 1–2 hrs followed by rinsing with shaking 10 times in deionized H_2_O and five times in MilliQ H_2_O. Slides were dried by centrifugation at 1,000 rpm for 5 min with a slide holder in a GS-6 Beckman centrifuge, and then were baked at 140°C in a vacuum oven for 4–5 hrs (Fisher Scientific Model 280A); (2) microarray preparation: each oligonucleotide probe was mixed with the Microarray Printing Solution (GenScript, Piscataway NJ) at a 1:5 ratio (v/v) to a final concentration of 50 μM in a well of a 384-well plate. Probes were then arrayed onto the washed glass slides with humidity between 50% and 55%, and temperature between 24.5°C to 26.5°C; (3) hybridization: Each glass slide with probe arrays was placed into a Corning slide cassette. Hybridization was performed in 30 μl of 1× hybridization solution (5× Denhart's solution, 0.5% SDS, 3 × SSC, 20 μl of ssDNA at 56°C for 2 hrs. The cassette was briefly soaked in iced water before opening. The slide was then washed with 1 × SSC and 0.1% SDS at 56°C for 10 min, rinsed twice with 0.5 × SSC for 30 sec and twice with 0.2 × SSC for 30 sec; and (4) probe labeling by single base extension: microarrays consisting of oligonucleotide probes were covered with 25 μl 1× labeling solution containing 20 units of Sequenase, 1× Sequenase buffer (GE Healthcare Life Sciences, Piscataway, NJ), and 750 nM Cy5-ddCTP (Applied Biosystems, Foster City, CA). The labeling reaction was performed at 70°C for 10 min. The slide was washed again under the same conditions used after hybridization.

### Microarray scanning and data analysis

Microarrays were scanned with a GenePix 4000 scanner (Axon Instruments, Foster City, CA). The resultant images were digitized with the accompanying software Genepix Pro (version 4.0). The mean values of the signals from the duplicate spots of each probe were used for the analysis in Tables 1 and 2. Background signal was determined by using negative control probes that were complementary to the intron sequences of the corresponding genes or random sequences, and was subtracted from the sample signals. For the comparative expression analysis of the cell lines MCF-7 and NCI/ADR-RES in Table 1, the array data were normalized by the Lowess smoothing method [45,46]. After background subtraction, genes with negative values of signal intensities in both duplicated samples were excluded for further analysis. The log ratios of the intensities of the remaining genes in two cells lines were used to make calls and to identify the differentially expressed genes in the samples.

## Authors' contributions

GH and QY designed and carried out the study, contributed to the probe design concept, and manuscript preparation. HL and GY contributed to the concept for designing primers. MA and HL contributed to the algorithm development in primer design for multiplexing amplification. HW contributed to the establishment of the microarray system. XC contributed to the establishment of the multiplex amplification. HL directed the study and contributed to manuscript preparation. All authors read and approved the final manuscript.

## Supplementary Material

Additional file 1 The 1,135 genes and probes used for expression profiling assay. The 1,135 genes included in the study and their names, chromosomal locations, probes, and their characteristics used for the high-throughput gene expression profiling of these genes. Click here for file

Additional file 2 Primers used for expression profiling of the 1,135 genes. primers and their characteristics used for the high-throughput gene expression profiling of the 1,135 genes. Click here for file

Additional file 3 Signal intensities from expression profiling of the 1,135 genes. Signal intensities from expression profiling of the 1,135 genes in the two cell lines MCF7 and NCI/ADR-RES and a single cell from NCI/ADR-RES. Click here for file
